# SMOX Inhibition Preserved Visual Acuity, Contrast Sensitivity, and Retinal Function and Reduced Neuro-Glial Injury in Mice During Prolonged Diabetes

**DOI:** 10.3390/cells13242049

**Published:** 2024-12-12

**Authors:** Moaddey Alfarhan, Fang Liu, Bayan R. Matani, Payaningal R. Somanath, S. Priya Narayanan

**Affiliations:** 1 Program in Clinical and Experimental Therapeutics, College of Pharmacy, University of Georgia, Augusta, GA 30907, USA; m.alfarhan@uga.edu (M.A.); fliu1@augusta.edu (F.L.); bmatani@augusta.edu (B.R.M.); sshenoy@augusta.edu (P.R.S.); 2 Research Division, Charlie Norwood VA Medical Center, Augusta, GA 30901, USA; 3 Culver Vision Discovery Institute, Augusta University, Augusta, GA 30907, USA; 4 Department of Clinical Practice, College of Pharmacy, Jazan University, Jazan 45142, Saudi Arabia

**Keywords:** diabetic retinopathy, neurodegeneration, glial injury, SMOX, MDL 72527, visual acuity, contrast sensitivity, electroretinography

## Abstract

Diabetic retinopathy, a major cause of vision loss, is characterized by neurovascular changes in the retina. The lack of effective treatments to preserve vision in diabetic patients remains a significant challenge. A previous study from our laboratory demonstrated that 12-week treatment with MDL 72527, a pharmacological inhibitor of spermine oxidase (SMOX, a critical regulator of polyamine metabolism), reduced neurodegeneration in diabetic mice. Utilizing the streptozotocin-induced diabetic mouse model and MDL 72527, the current study investigated the effectiveness of SMOX inhibition on the measures of vision impairment and neuro-glial injury following 24 weeks of diabetes. Reductions in visual acuity, contrast sensitivity, and inner retinal function in diabetic mice were improved by MDL 72527 treatment. Diabetes-induced changes in neuronal-specific class III tubulin (Tuj-1), synaptophysin, glutamine synthetase, and vimentin were attenuated in response to SMOX inhibition. In conclusion, our findings show that SMOX inhibition improved visual acuity, contrast sensitivity, and inner retinal function and mitigated diabetes-induced neuroglial damage during long-term diabetes. Targeting SMOX signaling may provide a potential strategy for reducing retinal neuronal damage and preserving vision in diabetes.

## 1. Introduction

Diabetic retinopathy (DR) is a complication of diabetes and the leading cause of vision loss in the working-age population worldwide [1]. According to the International Diabetes Federation (IDF), an estimated 537 million people are affected worldwide by diabetes, with this number expected to reach 783 million by 2045 [2]. The estimated number of patients with DR in 2020 was 103 million globally, and this figure is projected to rise to 160 million by 2045. It is anticipated that by 2045 around 50 million individuals will have vision-threatening DR [3]. Studies have shown that the prevalence of DR is higher in patients with type 1 diabetes (T1D) than in those with type 2 diabetes (T2D) [4]. While DR is recognized as a neurovascular disease, the current treatments primarily target vascular complications in the late stages of the disease and are associated with unfavorable side effects [5]. Therefore, there is an urgent need to identify novel therapeutic targets to preserve vision in diabetic patients.

Neurodegeneration is a hallmark of diabetic retinopathy [6,7]. Several reports have shown that retinal neurons become dysfunctional during the initial stages of diabetes [8,9,10,11,12,13]. Postmortem samples from diabetic patients have shown many apoptotic cells in the retinal layers, with higher levels in the ganglion cell layer (GCL) [14]. Studies using experimental models have also shown increased apoptotic cells in diabetic retinas, particularly within the GCL [8,9]. In addition, changes in electroretinography (ERG) occur at the early stages in diabetic patients [10,11,12,13]. Further, a reduction in visual function and changes in retinal structure have also been reported during the early stages of the disease [15,16,17,18,19].

Since retinal neuronal damage and dysfunction are recognized as early events in diabetes, targeting these neurons offers a promising therapeutic approach to preserving vision in diabetic patients. The mechanisms underlying neuronal degeneration and dysfunction in the diabetic retina are under extensive investigation, providing critical insights for developing strategies to prevent or halt the progression of DR. Many therapeutic efforts focusing on addressing oxidative stress, mitochondrial dysfunction, neurotrophic support, and stem cell-based interventions to protect retinal neurons are in progress [20,21,22,23]. However, the challenge remains in identifying therapeutic agents or targets that ensure sustained neuroprotection while minimizing adverse effects during long-term treatment. The dysregulation of polyamines and polyamine metabolism have been observed in different neurological diseases, such as Alzheimer’s disease [24], Parkinson’s disease [25], and traumatic brain injury [26]. Studies from our laboratory have demonstrated that the oxidation of polyamines is associated with neurovascular damage in the retina, and spermine oxidase (SMOX, an enzyme in polyamine catabolism) is critically associated with neuronal damage and dysfunction in retinal disease models [27,28,29,30]. SMOX expression is upregulated in the retinas of diabetic patients and diabetic mouse models [29,31]. A previous study from our laboratory demonstrated that the upregulation of SMOX in the retinas of streptozotocin (STZ)-diabetic mice (12 weeks post-induction) was associated with neurodegenerative changes such as retinal thinning and altered retinal function [29]. However, treatment with MDL 72527 (N1, N4-bis (2,3-butadienyl)-1,4-butane diamine), a pharmacological inhibitor of SMOX [32], improved these changes. Nevertheless, the effects of SMOX blockade during long-term diabetes and its impact on the measures of vision, which are needed to understand its translational impact, remain unclear. In the present study, using the STZ-treated experimental model of diabetes and MDL 72527 treatment, we investigated the effectiveness of SMOX inhibition on visual acuity, contrast sensitivity, inner retinal function, and neuro-glial injury 6 months post-diabetes.

## 2. Materials and Methods

### 2.1. Animals

In this study, we used 8- to 10-week-old male mice (C57BL6, Jackson Laboratories, Bar Harbor, ME, USA). All experimental procedures were performed following the ARVO Statement for the Use of Animals in Ophthalmic and Vision Research. All procedures were approved by the Institutional Animal Care and Use Committee of Augusta University, Augusta, GA, USA, and/or the Charlie Norwood VA Medical Center, Augusta, GA, USA. Every effort was made to minimize pain and animal suffering during the experimental procedures.

### 2.2. Induction of Diabetes and MDL 72527 Administration

The experimental model of type 1 diabetes was induced using streptozotocin (STZ) treatment as per the established methods [29]. Briefly, male C57BL/6 mice aged 8–10 weeks were treated with repeated intraperitoneal STZ injections (50 mg/kg/citrate buffer, up to five times). The control mice received citrate buffer injections. Animals with glucose levels of 350 mg/dL or higher were considered diabetic and maintained for 24 weeks post-diabetes onset.

**Figure 1:**
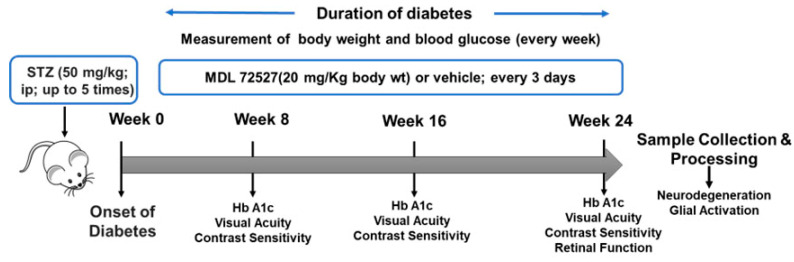
The experimental design showing the duration of diabetes and the time points of different treatments and measurements.

The mice were divided into four groups: vehicle-treated controls (Con + Veh), vehicle-treated diabetic mice (Db + Veh), MDL 72527-treated diabetic mice (Db + MDL), and MDL 72527-treated controls (Con + MDL). Mice in the Con + MDL and Db + MDL groups received 20 mg/kg of MDL 72527 in saline intraperitoneally (IP) every three days, starting immediately after the onset of diabetes and continuing for 24 weeks. Mice in the Con + Veh and Db + Veh groups received saline injections. The dose of 20 mg/kg every three days was based on our previous studies, which showed protective effects against diabetic retinopathy with no adverse effects [29]. A flow diagram with various treatments and experimental designs is presented as Figure 1.

### 2.3. Measurement of Blood Glucose and HbA1c

Blood glucose levels were measured weekly using the Alpha TRAK2 blood glucose monitoring system (Fisher Scientific, Pittsburgh, PA, USA). In addition, hemoglobin A1c was assessed every eight weeks using a Mouse Hemoglobin A1c (HbA1c) Assay Kit (Crystal Chem, Elk Grove Village, IL, USA).

### 2.4. Optokinetic Tracking Response

The mice were subjected to a virtual reality chamber to evaluate their visual behavior (OKT; Opto-Motry, Cerebral Mechanics, Medicine Hat, AB, Canada) available at the Augusta University Vision Core, according to the published methods [33,34]. These experiments were performed following 8, 16, and 24 weeks of diabetes. Briefly, a mouse was placed in the center of the arena, surrounded by four monitors. Vertical black and white waves were projected on the screens and moved at a speed of 12°/s. A contrast of 100% was used to measure spatial frequency (SF) thresholds. A staircase procedure was used in which the observer evaluated visual acuity from low to high. Contrast sensitivity was measured with an SF of 0.092; while the contrast started at 100%, it systematically reduced until the threshold was identified.

### 2.5. Electroretinography

ERG recordings were performed following 24 weeks of diabetes using the Touch/Touch feature of the Celeris Ophthalmic Electrophysiology System (Diagnosys, Lowell, MA, USA) available at the Augusta University Vision Core, according to the published methods [33,34]. The Touch/Touch protocol stimulates one eye at a time and uses the fellow, unstimulated eye as the reference. For scotopic ERGs, the mice were dark-adapted for approximately 16 h before the experiment and then tested using a series of light flashes of increasing energy (0.001, 0.005, 0.01, 0.1, 0.5, and 1.0 cd s/m^2^).

### 2.6. Western Blotting

Protein analysis using the Western blot method was performed according to the standardized methods in our laboratory [27,28,29,30]. Samples were homogenized in RIPA lysis buffer (Millipore, Billerica, MA, USA) containing a protease inhibitor cocktail (Thermo Scientific, West Columbia, SC, USA) and a phosphatase inhibitor cocktail (Thermo Scientific, West Columbia, SC, USA). The protein concentration was estimated using the BCA Protein Assay Kit (Thermo Scientific, West Columbia, SC, USA). Approximately 15 µg of protein samples was separated on an SDS-PAGE gel, transferred to a polyvinylidene difluoride (PVDF) membrane, and blocked in 5% non-fat dry milk in 1× Tris-buffered saline with 0.1% Tween (TBST). The membranes were incubated with the respective primary antibodies (Table 1). Next, the membranes were washed with TBST and then incubated with the respective secondary antibodies (anti-rabbit or anti-mouse HRP-conjugated secondary antibody) for one hour at room temperature. Protein bands were detected using enhanced chemiluminescence (ECL) (Thermo Fisher Scientific, Waltham, MA, USA) and the ChemiDoc Imaging System (Bio-Rad, Hercules, CA, USA). Band intensities were quantified using ImageJ software (version 1.54g) and normalized to the loading control.

### 2.7. Immunofluorescence Staining and Quantification

The immunostaining of retinal cryostat sections was performed according to the established methods in our laboratory [27,28,29]. Briefly, eyes were removed under deep anesthesia and fixed in 4% paraformaldehyde overnight at 4 °C and cryoprotected in 30% sucrose for 24 h. Cryostat sections (10 µm) obtained on glass slides were permeabilized and blocked in 10% normal donkey serum with 0.1% Triton X-100 for one hour. Next, the sections were incubated with primary antibodies (Table 1). Then, the sections were washed three times with PBS and incubated with fluorescein-conjugated secondary antibodies for 1–2 h at room temperature. Next, the sections were washed in PBS and covered with a mounting medium with DAPI (Vectashield; Vector Laboratories, Burlingame, CA, USA). Image acquisition was conducted using a confocal microscope (LSM 780; Carl Zeiss, Thornwood, NY, USA). Images were taken at a distance of 500 µm from the optic nerve, and the fluorescence intensity of neuronal or glial markers was quantified using ImageJ software. A minimum of three sections (20 µm apart) per retina were imaged and used for quantification studies.

### 2.8. Statistical Analysis

GraphPad Prism 9 was used for statistical analysis. Statistical analysis was performed using a one-way ANOVA followed by the Tukey test for multiple comparisons. Results were considered significant when *p* < 0.05.

## 3. Results

### 3.1. Blood Glucose and HbA1c Levels During Diabetes

**Figure 2:**
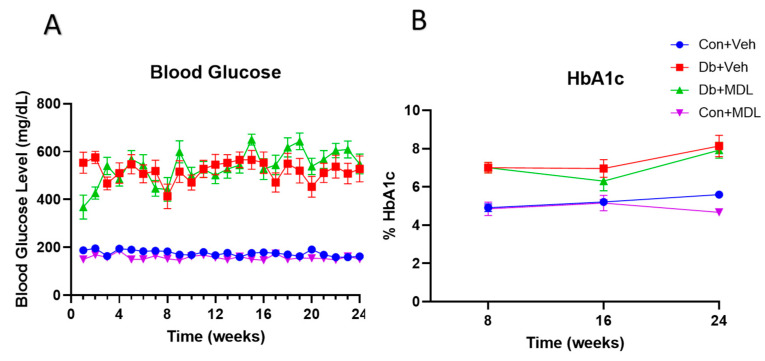
Changes in blood glucose and HbA1c during the treatment period. Changes in the blood glucose (**A**) were measured weekly, and those of the HbA1c were evaluated every 8 weeks (**B**). Data are presented as the mean ± SEM. N = 10–16 per group.

Blood glucose was measured weekly. Blood glucose levels remained elevated in diabetic mice during the study period (Figure 2A). HbA1c was measured every eight weeks. All the mice in both diabetic groups showed elevated HbA1c levels (Figure 2B). The measurements after the 24 weeks showed that mice in the diabetic groups had significantly higher blood glucose and HbA1c levels than the non-diabetic control groups (Table 2). The MDL 72527 treatment did not show any effect on the blood glucose levels or HbA1c in the diabetic mice (Table 2).

### 3.2. MDL 72527 Treatment Improved Visual Acuity and Contrast Sensitivity in Diabetic Mice

**Figure 3:**
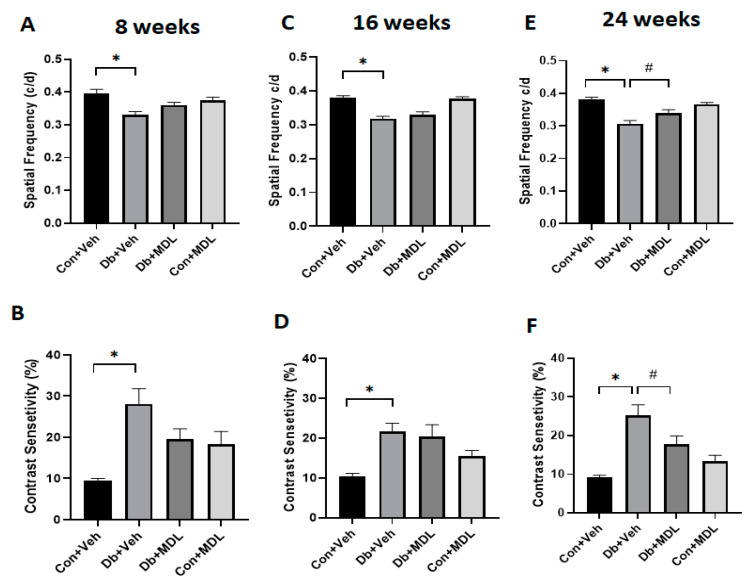
Changes in visual acuity and contrast sensitivity in response to SMOX inhibition. Optokinetic measurements showing changes in visual acuity and contrast sensitivity in the control and treatment groups following 8 weeks (**A**,**B**), 16 weeks (**C**,**D**), and 24 weeks of diabetes (**E**,**F**). Data are shown as the mean ± SEM. N = 10–16 per group. * *p* < 0.01; # *p* < 0.05.

Visual acuity (VA) and contrast sensitivity (CS) were assessed following 8, 16, and 24 weeks of diabetes onset at a speed of 12°/s. The mice at 8 and 16 weeks post-diabetes showed significant reductions in both VA (*p* < 0.01) and CS (*p* < 0.01) compared to the mice in the control group (Figure 3A–D). While the MDL 72527 treatment showed marked improvements in both VA and CS in the diabetic mice, these changes were not statistically significant compared to the vehicle-treated group. Visual acuity and contrast sensitivity were further considerably reduced in the vehicle-treated diabetic mice following 24 weeks of diabetes compared to the vehicle-treated control mice (*p* < 0.01) (Figure 3E,F). Upon the completion of 24 weeks of treatment, the mice in the MDL-treated diabetic group showed a significant improvement in visual acuity and contrast sensitivity (*p* < 0.05) compared to the vehicle-treated diabetic group (Figure 3E,F). These results suggest that SMOX inhibition reduced the loss of visual acuity and contrast sensitivity resulting from progressive neurodegeneration in the retinas during long-term diabetes. No noticeable changes were observed in the control mice that received the MDL 72527 treatment. The values for the confidence interval in these comparisons at the three time points that are presented in Supplementary Data S1.

### 3.3. Effect of SMOX Inhibition on ERG Responses

**Figure 4:**
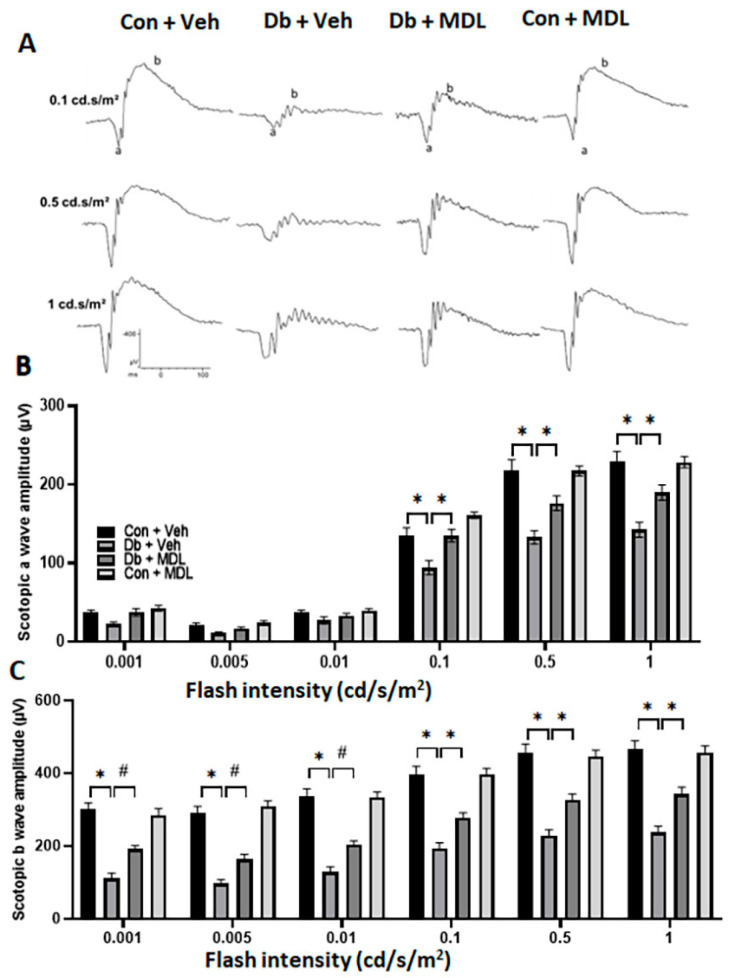
The effect of the MDL 72527 treatment on diabetes-induced functional changes in the retinas. Electroretinography studies demonstrating changes in scotopic ERG measurements performed following 24 weeks of diabetes. (**A**) Representative ERG tracings from four groups at various intensities. a, a-wave; b, b-wave. (**B**) Changes in scotopic a-wave amplitudes at various flash intensities and (**C**) b-wave amplitudes studied at flash intensities ranging from 0.001 to 1.0 cd/s/m^2^. Data are shown as the mean ± SEM. N = 9–12 per group. * *p* < 0.01; # *p* < 0.05.

ERG studies were performed 24 weeks following diabetes induction. Figure 4A indicates the representative tracings of ERG responses from the control and diabetic groups. Notable changes were not observed in the scotopic a-wave responses across the four groups at low light intensities (0.001, 0.005, and 0.01 cd/s/m^2^) (Figure 4B). With higher flash intensities (0.1, 0.5, and 1 cd/s/m^2^), the diabetic mice treated with the vehicle showed significantly lower amplitudes than the non-diabetic control mice (*p* < 0.01; Figure 4B). However, the treatment with MDL 72527 in the diabetic mice exhibited an improvement in the scotopic a-wave amplitude when compared to the vehicle-treated diabetic mice (*p* < 0.01; Figure 4B). The diabetic mice treated with the vehicle showed significant reductions in the scotopic b wave amplitude at all studied flash intensities (0.001, 0.005, 0.01, 0.1, 0.5, and 1 cd/s/m^2^) when compared to the control group (*p* < 0.01; Figure 4C). However, the MDL 72527 treatment of the diabetic mice exhibited a significant improvement in the scotopic b-wave amplitudes compared to the vehicle-treated diabetic mice at all intensities studied (Figure 4C). These data indicate that the treatment with MDL 72527 improved rod bipolar cells and rod function, which accounts for around 97% of photoreceptor cells [35], and improved inner retinal function. Responses from the mice in the control group that received the MDL 72527 treatment were similar to those of the vehicle-treated controls. The values for the confidence interval in these comparisons at all intensities are presented in Supplementary Data S1.

### 3.4. Effect of SMOX Inhibition on Neurodegeneration

**Figure 5:**
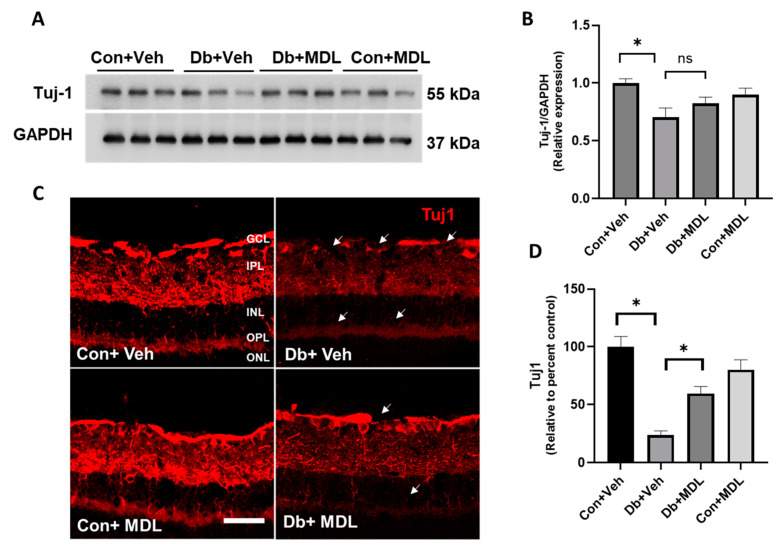
The effect of the MDL 72527 treatment on Tuj 1 levels following 24 weeks of diabetes. (**A**) Immunoblotting studies showing changes in the expression of Tuj-1 (neuron-specific beta-III tubulin) in diabetic retinas and the effect of the MDL 72527 treatment. (**B**) The quantification of intensity measurements demonstrating the changes in the diabetic retinas and the impact in response to the MDL 72527 treatment. (**C**) Representative confocal images showing the expression changes in Tuj-1 in the retinal sections of diabetic mice and their respective controls and the impact of SMOX inhibition. Arrows indicate areas with reduced Tuj 1 expression. (**D**) The quantification of fluorescence intensity measurements demonstrating the changes in Tuj 1 expression in the retinal sections and the impact in response to the MDL 72527 treatment. Data are presented as the mean ± SEM. (N = 5–6; * *p* < 0.01); ns, statistically non-significant. Scale bar: 50 μm. GCL, ganglion cell layer; IPL, inner plexiform layer; INL, inner nuclear layer.

Retinal neurons, including RGCs, are reported to be impacted by DR [36], and our previous studies have shown a reduction in the loss of RGCs in the diabetic retina [29]. In the present study, we further investigated changes in RGC neurons in the retinas following 24 weeks of diabetes. Tuj-1 is a neuronal marker expressed in RGCs and their axons [37,38,39]. In this study, we employed both immunoblotting and immunofluorescence staining experiments to assess the changes in Tuj-1 expression in all four groups following 24 weeks of diabetes. Results from the Western blot studies showed that Tuj-1 was significantly reduced in the diabetic retinas (*p* < 0.01) (Figure 4A,B). While the treatment with MDL 72527 improved Tuj-1 expression in the diabetic retinas, the changes were not statistically significant (Figure 5A,B). The MDL 72527 treatment did not show a difference between the two control groups. Immunostaining results further confirmed the significantly reduced Tuj-1 levels in the RGCs and their axons in the diabetic retinas compared to the controls (*p* < 0.01), which showed a significant improvement in response to SMOX inhibition (*p* < 0.01) (Figure 5C,D). The arrows in Figure 5C indicate areas with markedly reduced Tuj 1 levels. The values for the confidence interval in these comparisons are presented in Supplementary Data S1.

**Figure 6:**
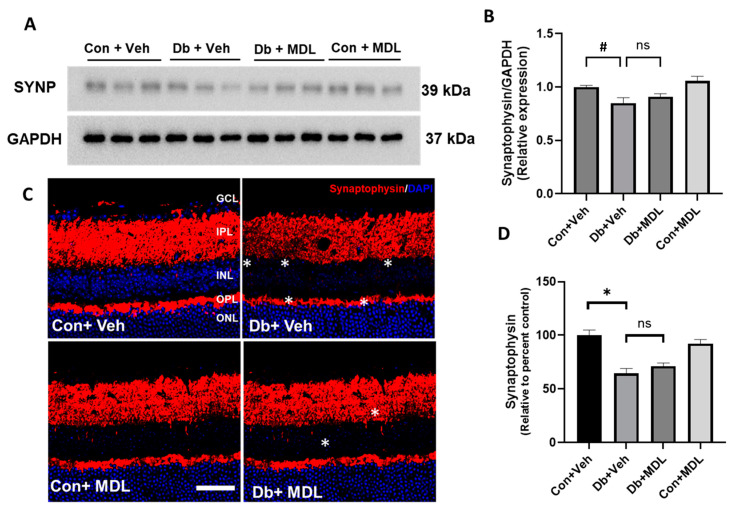
The effect of SMOX inhibition on synaptophysin expression. (**A**) Western blot studies showing changes in the expression of synaptophysin in the retinas of the diabetic mice and the impact of the MDL 72527 treatment. (**B**) The quantification of intensity measurements demonstrating the changes in synaptophysin expression in the diabetic retinas and the impact in response to the MDL 72527 treatment. (**C**) Representative confocal images showing the changes in the expression of synaptophysin in the retinal sections. Asterisks indicate areas with reduced synaptophysin expression. (**D**) The quantification of fluorescence intensity measurements demonstrating the changes in synaptophysin expression in the retinal sections and the impact in response to the MDL 72527 treatment. Data are presented as the mean ± SEM. (N = 6; * *p* < 0.01, # *p* < 0.05); ns, statistically non-significant. Scale bar: 50 μm. SYNP, Synaptophysin; IPL, inner plexiform layer; INL, inner nuclear layer; OPL, outer plexiform layer; ONL, outer nuclear layer.

Synaptophysin is a presynaptic protein associated with synaptic vesicles [40]. Synaptophysin, expressed in the inner and outer plexiform layers, is essential in forming and recycling synaptic vesicles [41,42,43,44,45]. Twenty-four weeks post-diabetes, the mice showed a significant reduction in the expression of synaptophysin in the retina when compared to the non-diabetic controls (*p* < 0.05) (Figure 6A,B). The diabetic mice treated with MDL 72527 showed an upregulation at the synaptophysin level. However, this was not statistically significant (Figure 5A,B). No significant difference in synaptophysin expression was observed between the control groups. Consistent with these results, the confocal images obtained from retinal cryostat sections immunostained with synaptophysin showed significantly reduced expression in the diabetic retinas compared to those in the control group (*p* < 0.01). While the MDL 72527 treatment improved synaptophysin in the diabetic retinas, the differences did not reach statistical significance (Figure 6C,D). The asterisks in Figure 6C indicate areas with reduced synaptophysin expression. The values for the confidence interval in these comparisons are presented in Supplementary Data S1.

### 3.5. Effect of SMOX Inhibition on Retinal Glial Damage

**Figure 7:**
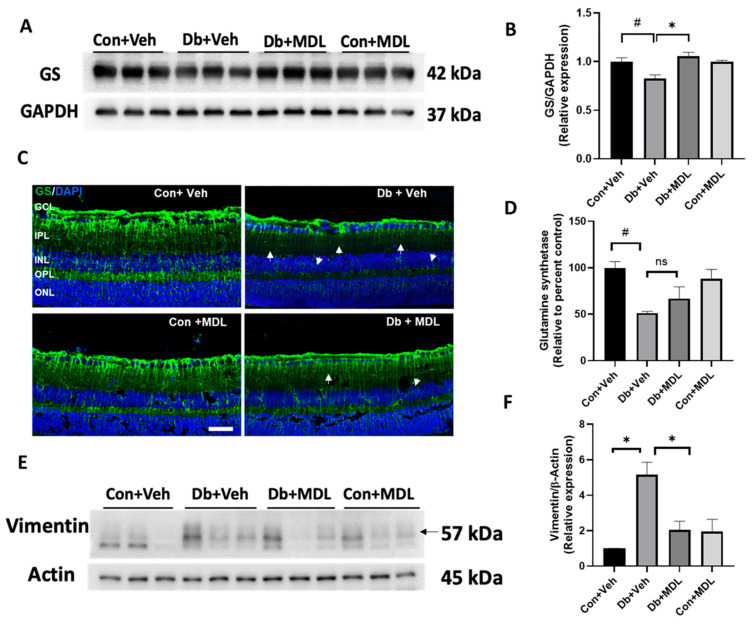
Changes in glial damage in the diabetic retinas in response to SMOX inhibition. (**A**,**B**) Western blot data showing the changes in the expression of GS in the retinas from the control and diabetic mice and changes in response to the MDL 72527 treatment. Data are presented as the mean ± SEM; N = 3; * *p* < 0.01; # *p* < 0.05. (**C**) Representative confocal images of the retinal sections showing the expression changes in glutamine synthetase in the diabetic retinas and the effect of the MDL 72527 treatment. Arrows indicate areas of reduced GS expression. (**D**) The quantification of fluorescence intensity measurements demonstrating the changes in GS expression in the retinal sections and the impact in response to the MDL 72527 treatment. Scale bar: 50 μm. GCL, ganglion cell layer; IPL, inner plexiform layer; INL, inner nuclear layer; OPL, outer plexiform layer; ONL, outer nuclear layer. ns, statistically non-significant. (**E**,**F**) Western blot data showing changes in the expression of vimentin in the diabetic retinas and the impact of SMOX inhibition. Data are shown as the mean ± SEM; N = 5–6. # *p* < 0.05; * *p* < 0.01.

Glial injury is a characteristic feature of the diabetic retina [46,47]. Glutamine synthetase (GS) and vimentin are markers of Müller cells [42]. GS is expressed in Müller cells, which convert neurotransmitters such as glutamate and gamma-aminobutyric acid (GABA) to glutamine [48]. In this study, the immunoblotting studies indicated a significant downregulation of the GS protein in the diabetic retinas from the vehicle-treated mice, compared to those of the non-diabetic controls (*p* < 0.01); this was reversed in the diabetic retinas from the MDL72527-treated group (*p* < 0.01) (Figure 7A,B). Representative images of the immunostained retinal sections show the downregulation of GS in the diabetic retinas (*p* < 0.01), which was improved in response to the MDL 72527 treatment. However, the differences did not reach statistical significance (Figure 7C,D). Arrows indicate areas with reduced GS expression in the diabetic retinal sections. No marked changes were evident in the control retinas. The values for the confidence interval in these comparisons are presented in Supplementary Data S1.

Vimentin is expressed in both astrocytes and Müller cells [49]. The expression of vimentin increases with retinal injury [44]. Our results showed that vimentin expression was significantly increased in the retinas of the diabetic mice in comparison to the mice in the vehicle-treated control group (Figure 7D). Interestingly, the treatment with MDL 72527 significantly decreased the diabetes-induced upregulation of vimentin expression in the retinas (*p* < 0.01) (Figure 7D,E). There was no significant difference between the non-diabetic groups. The values for the confidence interval in these comparisons are presented in Supplementary Data S1.

## 4. Discussion

A growing body of evidence compellingly indicates that retinal neuronal dysfunction occurs early in diabetes. Thus, it is crucial to identify therapeutic strategies that specifically target neuronal alterations to preserve vision in diabetic patients’ retinopathy and evaluate their long-term effects. Our prior studies established that SMOX expression significantly increases in the retina in models of ischemic retinopathy and excitotoxicity [28,30]. Notably, we demonstrated that the inhibition of SMOX using MDL 72527, a selective inhibitor for SMOX activity [50], protected against retinal neuronal death, retinal inflammation, and upregulated antioxidant signaling in various ocular disease models [27,28,29,30,51,52]. In our previous research using the STZ-induced model of diabetes, we found that 12-week treatment with MDL 72527 improved neuronal survival and retinal function in diabetic mice [29]. Building on this foundational work, the present study revealed that inhibition of SMOX preserved visual acuity, contrast sensitivity, and retinal function, which are the measures of vision impairment in diabetic patients, and reduced neuro-glial damage during prolonged diabetes. To the best of our knowledge, this is the first investigation of the effects of SMOX inhibition on diabetes-induced neurodegeneration and visual function during a long term. Our findings suggest that SMOX inhibition holds great promise as a therapeutic avenue for treating diabetic retinopathy in patients with this debilitating condition.

Clinical studies have unequivocally demonstrated that visual acuity and contrast sensitivity are significantly reduced in diabetic patients in stages of the disease, often preceding any detectable vascular changes [15,17,53,54]. In diabetic mice, a marked decline in visual acuity has been observed as early as four weeks post-diabetes, with this reduction persisting for up to 10 months [55,56]. Similarly, contrast sensitivity is also reduced in diabetic animal models [55,56,57,58]. Consistent with previous studies, our results from the present study revealed notable reductions in both visual acuity and contrast sensitivity in the diabetic mice across the three-time points examined. Importantly, we observed a significant decline in visual acuity and contrast sensitivity during the progression of diabetes. Interestingly, SMOX inhibition prevented the reduction in visual acuity and contrast sensitivity throughout, with the results being statistically significant at 24 weeks. These results are promising as they demonstrate the potential of SMOX inhibition in preserving vision during long-term diabetes. Another major characteristic of diabetic retinopathy is alterations in retinal function, which are often reflected in changes in ERG responses reported in diabetic retinopathy patients [10,11,12,13] and experimental models [29,59,60]. Our results corroborate the presence of these ERG alterations in diabetic mice. Furthermore, the treatment with MDL 72527 significantly improved scotopic responses in the diabetic mice following 24 weeks post-induction, suggesting a positive impact on photoreceptor and inner retinal neurons. Collectively, these results indicate the long-term beneficial effects of targeting SMOX for preserving visual function in the diabetic retina.

In the present study, we observed diabetes-induced changes in retinal neurons, which were studied by different markers. Tuj-1 is a reliable marker for RGCs and axonal integrity [39,61,62]. Notably, the expression of Tuj-1 was reduced in neuronal cells in response to high glucose and hydrogen peroxide exposure [62]. In line with previous experimental findings that demonstrated decreased Tuj1 levels in diabetic retinas [29,61], our results confirmed that long-term diabetes led to a significant decrease in Tuj-1 expression in the diabetic mice. Additionally, we investigated synaptophysin, a transmembrane protein found in presynaptic neurotransmitter vesicles that plays a crucial role in neurotransmitter release and recycling [63,64]. Notably, synaptophysin levels were reduced and decreased in the hippocampus of female diabetic mice, and the improvements in synaptophysin were linked to protection against cognition impairment [65]. Reduced synaptophysin levels were also reported to be associated with a decline in retinal function [66,67]. Our findings corroborate these observations, as we also identified a significant decrease in synaptophysin expression in the diabetic retinas. While the increases in Tuj-1 and synaptophysin levels following the MDL 72527 treatment were statistically non-significant, the observed upregulation underscores the potential of SMOX inhibition. The enhancement in these markers supports our hypothesis that MDL 72527 mitigates retinal neuronal damage resulting from long-term diabetes.

Our results indicate that the changes in Tuj-1 and synaptophysin expression in response to SMOX inhibition studied via the Western blotting of the retinal lysate were statistically non-significant. While analyses using fluorescent intensity measurements of images showed similar results for synaptophysin, the Tuj1 levels significantly improved in the diabetic retinas in the MDL 72527-treated mice. This could be resulting from the region-specific changes in the loss of RGCs and their axons in the diabetic retinas. While the Western blot studies utilized whole retinal lysates, the section images were from midcentral retinas. This could also be a result of the changes in RGC densities in central and peripheral retinas [68] and the expression of Tuj1 and synaptophysin in neurons other than RGCs. While Tuj1 is considered a marker for RGCs and their axons, it is also expressed by other retinal neurons [69]. Similarly, synaptophysin is expressed by bipolar cells as well [70,71]. The treatment with MDL 72527 may also create a neuroprotective microenvironment by reducing gliosis and improving glutamate metabolism, which indicates a glial-centered therapeutic effect.

Injury to glial cells is a hallmark of the diabetic retina [46]. Müller cells, as the primary glial cells, play critical roles in preserving retinal integrity, maintaining homeostasis, and providing neuroprotection [72]. Glutamine synthetase (GS) expressed in Müller cells converts glutamate to glutamine, a less active metabolite [73]. This enzymatic action protects against elevated glutamate levels and retinal excitotoxicity [74,75]. A previous investigation showed that the retinas of diabetic mice exhibited reduced mRNA and the protein expression of GS and that the extent of this reduction worsened with the duration of diabetes. Furthermore, this reduction in GS was associated with deterioration in visual function [76]. Our findings align with these observations, confirming reduced GS levels in the diabetic retinas. Further, SMOX inhibition improved GS levels in the diabetic retinas, suggesting the role of the antioxidant defense system. The differences observed between the Western blot and immunostaining using GS in our study could have resulted from region-specific differences in GS expression in the retinas. We previously reported the impact of SMOX inhibition on preserving antioxidant defense in retinal excitotoxicity [51]. Another marker for glial cell activation is vimentin, which is expressed in both Müller cells and astrocytes [77]. Experimental models of diabetes have demonstrated a significant increase in vimentin expression following 2 to 6 months of diabetes [55,77]. This increase aligns with our findings of elevated vimentin levels in the diabetic retinas. Vimentin has been implicated in photoreceptor degeneration following retinal detachment in mice [49]. Notably, our studies revealed reduced vimentin levels in response to SMOX inhibition, further supporting its protective role against glial damage in the diabetic retina. While we were unable to examine changes in vimentin expression in diabetic retinas through immunofluorescence, the data we gathered provided compelling evidence of the impact of SMOX inhibition on glial cell health in the context of diabetes.

SMOX activation is recognized as a primary source of endogenous acrolein, a highly reactive aldehyde known for its potent oxidative damage [78]. Acrolein exerts toxic effects on various cell types, causing significant cellular injury by leading to DNA and protein adduction, increasing oxidative stress, and disrupting mitochondrial function and cellular membranes [79]. Its formation and accumulation have been implicated in several neurodegenerative conditions, including spinal cord injury [80,81,82], brain infarction [83,84], Alzheimer’s disease [85], and multiple sclerosis [86,87,88]. Previous studies from our lab have demonstrated an increase in conjugated acrolein in GCL and the inner nuclear layer (INL). This elevation correlates with retinal neuronal damage in experimental models of diabetic retinopathy [29]. Importantly, treatment with MDL 72527 significantly reduced the formation of acrolein conjugates and mitigated retinal neuronal damage in both diabetes and multiple sclerosis models [29,52]. Furthermore, a prior study indicated that Müller cells exposed to hypoxic conditions in vitro produced H2O2 and acrolein, which were reduced by treatment with MDL 72527 [31]. Acrolein exposure has been shown to increase reactive oxygen species formation and promote glial migration in Müller cells [88,89]. Similarly, microglial exposure to acrolein enhances cell migration, as well as cyclooxygenase 2 (COX-II) and ROS production [51,90]. In an in vivo study using STZ-treated diabetic rats, the administration of an acrolein scavenger for three months led to decreased glial cell activation and improved retinal function [91]. Our previous work also indicated that the inhibition of SMOX using MDL 72527 reduces glial activation in a model of retinal excitotoxicity [28,51]. These findings collectively suggest that lowering acrolein conjugates can effectively protect against retinal glial cell activation. In the current study, the MDL 72527 treatment reduced vimentin levels and upregulated the expression of GS in the diabetic retinas. Together, these observations, along with the improvements in ERG responses, indicate that long-term SMOX inhibition offers significant protection against retinal neuroglial damage in diabetic mice. However, this study comes with some limitations as well. While both type 1 and type 2 diabetes contribute to vision loss, the majority of diabetic patients suffer from T2D. However, our present study investigated the impact of SMOX inhibition using only one model of type 1 diabetes. Further, for intervening SMOX function, the present study did not incorporate other approaches beyond pharmacological inhibition. Our future studies will employ experimental models of both types of diabetes.

## 5. Conclusions

In conclusion, our study demonstrates that pharmacological inhibition of SMOX improved visual acuity, contrast sensitivity, retinal function, and reduced neuro-glial injury during a prolonged period of disease in an experimental model of type 1 diabetes. These findings suggest that early intervention via targeting SMOX signaling represents a promising new strategy for mitigating vision loss associated with diabetes, offering hope for improved outcomes in affected patients.

## Figures and Tables

**Table 1:** Antibodies used in the study.

Antibody	Dilution	Experiment	Catalog Number	Company
Actin	1:1000	Western blot	4970S	Cell Signaling Technology, Danvers, MA, USA
GAPDH	1:1000	Western blot	5174S	Cell Signaling Technology, Danvers, MA, USA
Glutamine synthetase	1:200	Immunostaining	GTX630654	GeneTex, Irvine, CA, USA
Glutamine synthetase	1:10,000	Western blot	GTX630654	GeneTex, Irvine, CA, USA
Synaptophysin	1:200	Immunostaining	MABN1193	EMD Millipore, Burlington MA, USA
Synaptophysin	1:5000	Western blot	MABN1193	EMD Millipore, Burlington, MA, USA
Tuj-1	1:200	Immunostaining	MAB1195	R&D Systems, Minneapolis, MN, USA
Tuj-1	1:4000	Western blot	MAB1195	R&D Systems
Vimentin	1:1000	Western blot	M072529-2	Agilent Technologies, Santa Clara, CA, USA

**Table 2:** Changes in blood glucose and HbA1c following 6 months of diabetes. Data are represented as the mean ± SD. Compared to the control + vehicle, both diabetic groups showed a significant increase in the levels of blood glucose and glycated hemoglobin (HbA1c). (* *p* < 0.01; N = 10–15 per group).

Group	Blood Glucose (mg/dL)	HbA1c (%)
Control + Vehicle	163 ± 11	5.6 ± 0.76
Diabetic + Vehicle	527 ± 210 *	8.1 ± 2.2 *
Diabetic + MDL	549 ± 150 *	7.9 ± 1.5 *
Control + MDL	151 ± 11	4.7 ± 0.5

## Data Availability

All the data are included in the manuscript.

## Supplementary Materials

The following supporting information can be downloaded at: https://www.mdpi.com/article/10.3390/cells13242049/s1, Data S1: The values for the confidence interval in the group comparisons of visual acuity (A), contrast sensitivity (B) ERG responses (C), Tuj1 (D), Synaptophysin (E), Glutamine Synthetase (F) and Vimentin (G) are presented.
