# Supplementary material for: SMOX Inhibition Preserved Visual Acuity, Contrast Sensitivity, and Retinal Function and Reduced Neuro-Glial Injury in Mice During Prolonged Diabetes
Source: Cells. 2024 Dec 12;13(24):2049. doi: 10.3390/cells13242049 (PMC11674681; doi:10.3390/cells13242049)
Supplement: Supplementary file 1 [file cells-13-02049-s001.zip › cells-3319121-supplementary.pdf]

Data S1. The values for the confidence interval in the group comparisons of visual acuity (A), contrast sensitivity (B) ERG responses (C), Tuj1 (D), Synaptophysin (E), Glutamine Synthetase (F) and Vimentin (G) are presented.

A. Visual Acuity (VA)

**8 Weeks**	**Mean Diff.**	**95.00% CI of diff.**	**Adjusted P Value**
Con +Veh vs. Db +Veh	0.06416	0.02835 to 0.09996	<0.0001
Db +Veh vs. DB+MDL	-0.02953	-0.06595 to 0.006883	0.1507
**16 Weeks**			
Con +Veh vs. Db +Veh	0.06083	0.03506 to 0.08661	<0.0001
Db +Veh vs. Db +MDL	-0.01187	-0.03805 to 0.01432	0.6279
**24 Weeks**			
Con +Veh vs. Db +Veh	0.0749	0.04576 to 0.1040	<0.0001
Db +Veh vs. Db +MDL	-0.03337	-0.06361 to -0.003129	0.0253

B. <u>Contrast Sensitivity (CS)</u>

**8 Weeks**	**Mean Diff.**	**95.00% CI of diff.**	**Adjusted P Value**
Con +Veh vs. Db +Veh	-18.63	-28.24 to -9.023	<0.0001
Db +Veh vs. Db +MDL	8.523	-1.083 to 18.13	0.0989
**16 weeks**			
Con +Veh vs. Db +Veh	-11.33	-19.08 to -3.591	0.0016
Db +Veh vs. Db +MDL	1.267	-6.476 to 9.009	0.9722
**24 weeks**			
Con +Veh vs. Db +Veh	-16.16	-23.11 to -9.210	<0.0001
Db +Veh vs. Db +MDL	7.598	0.2723 to 14.92	0.0394

C. ERG Responses

	Mean diff	95.00% CI of diff.	Adjusted P Value
**0.1 cd/s/m2**			
Con + Veh vs. Db + Veh	41.05	15.82 to 66.28	0.0002
Db + Veh vs. Db + MDL	-40.89	-67.30 to -14.48	0.0005
**0.5 cd/s/m2**			
Con + Veh vs. Db + Veh	85.59	60.36 to 110.8	<0.0001
Db + Veh vs. Db + MDL	-43.42	-69.82 to -17.01	0.0002
**1.0 cd/s/m2**			
Con + Veh vs. Db + Veh	86.57	61.34 to 111.8	<0.0001
Db + Veh vs. Db + MDL	-47.12	-73.53 to -20.71	<0.0001

D. <u>Tuj 1</u>

Western blot	Mean Diff.	95.00% CI of diff.	Adjusted P Value
Con +Veh vs. Db +Veh	0.2971	0.06388 to 0.5302	0.0098
Db +Veh vs. Db +MDL	-0.1209	-0.3541 to 0.1123	0.4807

Immunostaining	Mean Diff.	95.00% CI of diff.	Adjusted P Value
Con + Veh vs. Db + Veh	76.37	48.76 to 104.0	<0.0001
Db + Veh vs. Db + MDL	-35.91	-63.52 to -8.303	0.0083

E. <u>Synaptophysin</u>

Western blot	Mean Diff.	95.00% CI of diff.	Adjusted P Value
Con + Veh vs. Db + Veh	0.1511	0.0008957 to 0.3012	0.0483
Db + Veh vs. Db + MDL	-0.0611	-0.2113 to 0.08906	0.6705

Immunostaining	Mean Diff.	95.00% CI of diff.	Adjusted P Value
Con + Veh vs. Db + Veh	35.62	19.39 to 51.85	<0.0001
Db + Veh vs. D b+ MDL	-6.787	-23.02 to 9.444	0.6517

F. <u>Glutamine Synthetase</u>

Western blot	Mean Diff.	95.00% CI of diff.	Adjusted P Value
Con + Veh vs. Db + Veh	0.1700	0.01713 to 0.3229	0.0303
Db + Veh vs. Db + MDL	0.2256	-0.3785 to -0.07270	0.0293

Immunostaining	Mean Diff.	95.00% CI of diff.	Adjusted P Value
Con + Veh vs. Db + Veh	48.65	2.515 to 94.78	0.0363
Db + Veh vs. Db + MDL	-15.64	-57.22 to 25.94	0.7256

G. <u>Vimentin</u>

Western blot	Mean Diff.	95.00% CI of diff.	Adjusted P Value
Con +Veh vs. Db + Veh	-4.153	-6.404 to -1.902	0.0003
Db + Veh vs. Db + MDL	3.114	0.8627 to 5.364	0.0054
